# Post-Ischemic Treatment of Recombinant Human Secretory Leukocyte Protease Inhibitor (rhSLPI) Reduced Myocardial Ischemia/Reperfusion Injury

**DOI:** 10.3390/biomedicines9040422

**Published:** 2021-04-13

**Authors:** Podsawee Mongkolpathumrat, Anusak Kijtawornrat, Eakkapote Prompunt, Aussara Panya, Nipon Chattipakorn, Stephanie Barrère-Lemaire, Sarawut Kumphune

**Affiliations:** 1 Graduate Programs in Biomedical Sciences, Faculty of Allied Health Sciences, Naresuan University, Phitsanulok 65000, Thailand; podsaweem61@nu.ac.th; 2 Integrative Biomedical Research Unit (IBRU), Faculty of Allied Health Sciences, Naresuan University, Phitsanulok 65000, Thailand; 3 Department of Physiology, Faculty of Veterinary Science, Chulalongkorn University, Bangkok 10330, Thailand; anusak.k@chula.ac.th; 4 Unit of Excellence in Infectious Disease, Department of Medical Technology, School of Allied Health Sciences, University of Phayao, Phayao 56000, Thailand; eakkapote.pr@up.ac.th; 5 Department of Biology, Faculty of Science, Chiang Mai University, Chiang Mai 50200, Thailand; aussara.pan@cmu.ac.th; 6 Cardiac Electrophysiology Research and Training Centre, Faculty of Medicine, Chiang Mai University, Chiang Mai 50200, Thailand; nchattip@gmail.com; 7 Institut de Génomique Fonctionnelle, Université de Montpellier, CNRS, Inserm, 141, rue de la Cardonille, 34094 Montpellier, France; Stephanie.Barrere@igf.cnrs.fr; 8 Biomedical Engineering Institute (BMEI), Chiang Mai University, Chiang Mai 50200, Thailand

**Keywords:** ischemic heart disease, ischemia/reperfusion injury, secretory leukocyte protease inhibitor (SLPI), cardioprotection, post-ischemic treatment

## Abstract

Myocardial ischemia/reperfusion (I/R) injury is a major cause of mortality and morbidity worldwide. Among factors contributing to I/R injury, proteolytic enzymes could also cause cellular injury, expand the injured area and induce inflammation, which then lead to cardiac dysfunction. Therefore, protease inhibition seems to provide therapeutic benefits. Previous studies showed the cardioprotective effect of secretory leukocyte protease inhibitor (SLPI) against myocardial I/R injury. However, the effect of a post-ischemic treatment with SLPI in an in vivo I/R model has never been investigated. In the present study, recombinant human (rh) SLPI (rhSLPI) was systemically injected during coronary artery occlusion or at the onset of reperfusion. The results show that post-ischemic treatment with rhSLPI could significantly reduce infarct size, Lactate Dehydrogenase (LDH) and Creatine kinase-MB (CK-MB) activity, inflammatory cytokines and protein carbonyl levels, as well as improving cardiac function. The cardioprotective effect of rhSLPI is associated with the attenuation of p38 MAPK phosphorylation, Bax, caspase-3 and -8 protein levels and enhancement of pro-survival kinase Akt and ERK1/2 phosphorylation. In summary, this is the first report showing the cardioprotective effects against myocardial I/R injury of post-ischemic treatments with rhSLPI in vivo. Thus, these results suggest that SLPI could be used as a novel therapeutic strategy to reduce myocardial I/R injury.

## 1. Introduction

Impaired blood supply in cardiac tissue due to coronary occlusion, particularly during acute myocardial infarction (AMI), leads to cardiac tissue injury or death, which subsequently causes cardiac dysfunction and mortality. The most effective therapeutic intervention for limiting the aggravation of tissue injury and death is to restore blood flow using reperfusion therapy. This could be performed by thrombolytic treatment or percutaneous coronary intervention (PCI) [1]. Reperfusion acts as a “double-edged sword” due to the fact that reperfusion can also extend myocardial injury [2] due to a rapid restoration of physiological pH, a burst of oxidative stress, intracellular and mitochondrial calcium overload, opening of the mitochondrial permeability transition pore (MPTP) and cardiac tissue inflammation [3]. Altogether, the detrimental consequences of reperfusion are referred to as reperfusion injury. Therefore, any post-ischemic interventions or treatments that prevent the detrimental activation of reperfusion injury are likely to decrease infarct size, save patients’ lives and improve their quality of life.

During myocardial ischemia and reperfusion, various biochemical processes contribute to tissue damage, cell necrosis and subsequent cardiac function impairment [4,5]. Those include post-ischemic inflammation, production of oxygen radicals, polymorphonuclear cell infiltration and protease release. Protease enzymes cause widespread destruction and have relatively long half-lives in tissue [6]. Moreover, an increase in proteolytic activity by chymase, matrix metalloproteinases, calpains, cathepsins and caspases contributes to myocardial infarction and cardiac remodeling, which is a major mechanism for the progression of heart failure [7]. Therefore, the idea of using protease inhibitors could possibly be an alternative therapeutic strategy to decrease myocardial I/R injury and improve morbidity/mortality in AMI patients.

Secretory leukocyte protease inhibitor (SLPI) is an 11.7 kDa non-glycosylated cationic protein, which has been reported to inhibit several inflammatory proteases [8], reduce inflammation [9], provide an antimicrobial effect [10], inhibit HIV infection [11], increase the levels of antioxidants [12] and reduce cellular apoptosis [13]. In recent years, several studies highlighted the cardioprotective effect of SLPI, which could also be a novel candidate for myocardial I/R injury treatment. In 2008, Schneeberger et al. reported that recombinant human (rh) SLPI (rhSLPI) could inhibit protease enzymes and post-ischemic inflammation in cardiac transplant [14]. More intensive investigation from our previous studies showed the cardioprotective effect of SLPI against I/R injury ex vivo and in vitro in cardiomyocytes, cardiac fibroblasts and vascular endothelial cells [3,15,16,17,18]. In addition, secretion of endothelial-derived rhSLPI could protect cardiomyocytes from I/R injury [19], while the rhSLPI protein itself could enhance the preservation of vessel grafts [3].

Our previous findings were obtained with a pre-treatment with rhSLPI, prior to the ischemic insult. However, since myocardial infarction in the actual clinical setting is unpredictable, rhSLPI should be evaluated as a postconditioning treatment, such as after the ischemic period or at the onset of reperfusion. More importantly, most of the previous findings were demonstrated in an in vitro and ex vivo study, whereas the effect of rhSLPI in an in vivo model has not been intensively investigated.

In the present study, we determined the effects of rhSLPI on physiological and biological effects when administered after coronary occlusion in an in vivo rat model of ischemia/reperfusion injury.

## 2. Materials and Methods

### 2.1. Experimental Animal and Ethical Approval

The animal experiments in this study were conducted according to the Guidance on the Operation of the Animals (Scientific Procedures) Act 1986 and the World Health Organization Guidelines for Breeding and Care of Laboratory Animals. All the protocols were approved by the committee of the Center for Animal Research, Naresuan University (NU-AE620615) and the Chulalongkorn University Laboratory Animal Center (CULAC), Protocol No.1973025. All procedures were designed to minimize the pain, suffering and distress of the animals involved.

Fifty-six adult male Wistar rats (8 weeks old) weighing 200−250 g (*n* = 14/group, 8 for physiology and 6 for biochemical study) in this study were purchased from Nomura Siam International, Bangkok, Thailand. They were maintained in an environmentally controlled condition (22 °C ± 1 °C, 12:12 h light/dark cycle) at the Chulalongkorn University Laboratory Animal Center (CULAC), Bangkok, Thailand.

### 2.2. Experimental Groups

**Figure 1:**
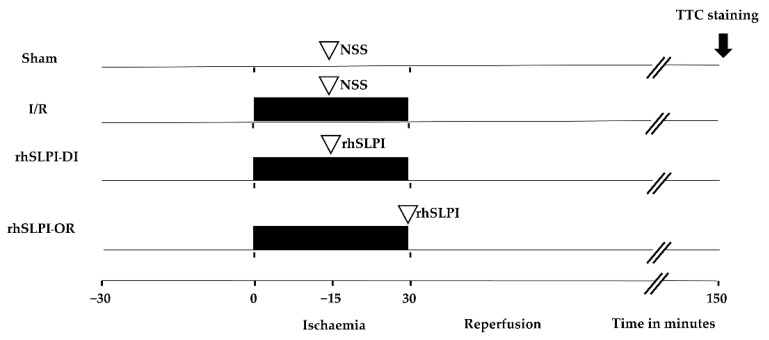
Schematic diagram of experimental protocol.

Fifty-six adult male Wistar rats were randomly allocated into 4 groups as illustrated in Figure 1. In the sham group, surgery was performed without left anterior descending (LAD) coronary artery ligation. In the I/R group, rats were subjected to a 30-min LAD occlusion followed by a 120-min reperfusion and were assigned to receive vehicle (normal saline solution). The fifty micrograms of recombinant human SLPI (rhSLPI), which was purchased from Sino Biology Inc. (Beijing, China), was administered by intravenous injection 15 min after the coronary ligation during ischemia (rhSLPI-DI) or at onset of reperfusion (rhSLPI-OR).

### 2.3. Surgical Preparation of Myocardial I/R Model in Rats

Rats were anesthetized in an isoflurane induction chamber followed by tracheotomy for intubation and connected to the rodent ventilator (VentElite, Harvard apparatus, MA, USA). The tidal volume was set at 2.5 mL with a rate of 80 breaths/min. A level of anesthesia was maintained with isoflurane 1.8−2.0% in 100% oxygen. The body temperature was maintained at 37 °C using a warm water heating pump. The lead II electrocardiogram (ECG) was attached to the rat and connected to the ECG amplifier. The carotid artery was cannulated with a Mikro-Tip^®^ pressure catheter for rat (Millar, Houston, TX, USA) for measuring left ventricular pressure (LVP) parameters. The jugular vein was also cannulated with a fluid-filled catheter for blood collection and rhSLPI administration. Then, left thoracotomy was performed to reveal the left coronary artery. A 6−0 synthetic, monofilament, nonabsorbable polypropylene suture was tied around the left anterior descending (LAD) of the left main coronary artery with the PE-50 tube to simulate the ischemic condition. Regional ischemia was confirmed with an elevation of the ST-segment in lead II tracing and faded whitish color tissue by visual inspection. The ligation was released after 30 min of ischemia, and the ligation was released by pulling the tube out of the ligation while the suture remained at the same place. The reperfusion was continued for 120 min. The ECG and LVP were recorded throughout the experiment with an IOX2 data acquisition & analysis software system (IOX 2.10.8.6, EMKA Technologies, Paris, France) and were stored on a hard drive for later analysis with ECG Auto software (ECG Auto 3.5.5.12, EMKA Technologies, Paris, France). The LVP was analyzed for end-diastolic pressure (EDP), end-systolic pressure (ESP), develop pressure (devP) and heart rate (HR). The contractility was inferred from the maximum rate of rise in the LVP (dP/dtmax) and contractility index (CI) defined as the dP/dtmax over the pressure at that point. In addition, the lusitropic indices were determined by the maximum rate of fall in the LVP (dP/dtmin) and Tau. At the end of the protocol, the hearts were quickly excised for infarct size assessment.

### 2.4. Evaluation of Heart Weight, Infarct Size and Area at Risk

After the end of the reperfusion protocol, the LAD was re-occluded and 2% (*w*/*v*) Evans blue solution (in PBS) was intravenously injected in order to differentiate the area at risk (non-perfused during the coronary occlusion) from the non-ischemic area. At the end of the surgical protocol, the heart was excised, weighed and transversally sliced in 1-mm-thick slices that were subsequently incubated in 1% (*w*/*v*) 2,3,5-triphenyl tetrazolium chloride (TTC) at 37 °C for 10 min to define the necrotic myocardium after enhancing the contrast between stained and unstained TTC tissues with a 10% (*v*/*v*) formalin (15−20 h) final incubation. The infarct area (TTC-negative) and the non-ischemic area (Evans blue-stained area) were measured by using ImageJ software.

### 2.5. Determination of Serum Creatine Kinase (MB Isoenzyme) and Lactate Dehydrogenase (LDH) Activity

Serum was collected at the end of the study protocol for determining the level of cardiac biomarkers. The creatine kinase (MB isoenzyme) and lactate dehydrogenase (LDH) activity was analyzed by using an automated biochemistry analyzer (Cobas c 111 analysers, Roche, Basel, Switzerland).

### 2.6. Heart Tissue Homogenization and Protein Collection

Another set of experiments was performed on another group of rats to assess the biochemical parameters. At the end of the I/R protocol, the heart was rapidly excised, and then the whole ventricular tissue was collected, quickly frozen in liquid nitrogen and stored at −80 °C until analysis. One hundred milligrams of tissue from each organ was isolated and then homogenized by a hand homogenizer on ice. One hundred milligrams of heart tissue was homogenized with 1 mL of homogenization buffer (20 mM Tris HCl pH6.8, 1 mM Na_3_VO_4_, 5 mM NaF and cOmplete™ Protease Inhibitor Cocktail). The tissue homogenate (100 mg/mL tissue extract) was centrifuged at 14,000 rpm for 10 min at 4 °C. The supernatants were collected and frozen for further analysis.

### 2.7. Determination of Protein Concentration by Bradford Assay

Determination of protein concentration was performed by using Bradford reagent (HIMEDIA^®^). Five microliters of protein extractions was added into 250 µL of Bradford reagent and mixed by pipetting. The reactions were incubated at room temperature for at least 5 min and the absorbance was measured at λ595 nm by a spectrophotometer. The protein concentration was determined by using bovine serum albumin (BSA) as a standard protein for calculating and normalizing the amount of total protein that was used in the ELISA method and Western blot analysis at 100 μg and 25 µg, respectively.

### 2.8. Determination of Inflammatory Cytokines Level by Enzyme-Linked Immunosorbent Assay (ELISA)

The ELISA (ABTS ELISA Buffer Kit, Peprotech^®^) was prepared at room temperature by gentle mixing. Firstly, the 96-well plate was pre-coated by adding 100 μL of 1 μg/mL capture antibody to each well and incubated overnight at room temperature. On the next day, the plate was inverted for removing the liquid and blotted on a paper towel. After that, the plate was washed with 200 μL washing buffer solution 4 times. The blocking solution was added into the well at 200 μL and incubated for 1 h. The reactions were washed 4 times again. One hundred micrograms of tissue homogenates was added into the wells and incubated at room temperature for at least 2 h. The plate was washed again and 100 μL of detection antibody was added and incubated at the room temperature for 2 h. The plate was washed again and 100 μL of Avidin-HRP conjugated 1:2000 was added and incubated at room temperature for 30 min. Then, the plate was washed and 100 μL of ABTS liquid substrate was added into the well and incubated at room temperature for color development. The sample absorbance was measured by a spectrophotometer at 405 nm with wavelength correction set at 650 nm.

### 2.9. Spectrophotometric Determination of Protein Carbonyl (PC) Content Level

Oxidative induction is a key factor leading to cardiac cell death in myocardial ischemia and ischemia/reperfusion injury [20] and subsequently results in biochemical modifications of some biomolecules such as proteins, lipids and DNA [21]. Ischemia-modified albumin (IMA) and protein carbonyl (PC) are proteins that are considered as a modified product on the similar basis of oxidative stress-induced protein modification and may have diagnostic potential in acute myocardial infarction [22]. In this study, the oxidatively modified proteins including IMA and PC were determined in both serum and cardiac tissue homogenate.

Both serum and cardiac tissue protein carbonyl (PC) content levels were spectrophotometrically determined by colorimetric DNPH assay, as previously described [23]. Firstly, protein extracts were diluted at 1:10 with phosphate-buffered saline (PBS). Two hundred microliters of diluted protein extractions was added into 800 µl of 10 mM DNPH in 2.5 M HCl. One milliliter of 20% (*w*/*v*) trichloroacetic acid (TCA) was then added and centrifuged at 10,000× *g* for 10 min at 4 °C to precipitate the protein. The protein pellet was washed 3 times with 1 mL of 1:1 (*v*/*v*) ethanol/ethyl acetate and centrifuged at 10,000× *g* for 10 min at 4 °C. After final washing, the protein pellet was resuspended in 500 µL of 6 M guanidine hydrochloride and centrifuged at 10,000× *g* for 10 min at 4 °C. The supernatant was collected and the absorbance was measured at 370 nm using 6 M guanidine hydrochloride as a blank. The PC content (nmol/mg) was calculated following the procedure used in a previous study [23].

### 2.10. Spectrophotometric Determination of Ischemia-Modified Albumin (IMA) Level

Determination of ischemia-modified albumin was performed by using albumin cobalt binding (ACB) assay, as previously described [23]. Two hundred microliters of serum or cardiac tissue homogenate was added into 50 μL of 0.1% (*w*/*v*) Cobalt chloride and mixed gently. The samples were incubated at room temperature for 10 min for adequate cobalt–albumin binding. Then, fifty microliters of 1.5 mg/mL dithiothreitol (DTT) was added to colorize the reaction and incubated at room temperature for 2 min. One milliliter of 0.9% (*w*/*v*) sodium chloride (NaCl) was added to quench the reaction and absorbance was measured at 470 nm. The determination of ischemia-modified albumin was calculated by serum cobalt blank without DTT as a blank of individual samples.

### 2.11. Western Blot Analysis

The heart homogenate was mixed with an equal volume of 2×SDS-PAGE sample buffers, containing 10% (*v*/*v*) 2-mercaptoethanol and bromophenol blue dye. The sample was boiled for 10 min. A measured 25 µg of protein was loaded into SDS-PAGE polyacrylamide gel electrophoresis gel for separation. Electrophoresis was performed at 120 V for 2 h. After separation, proteins were transferred to Immobilon-P membranes (Millipore) using a semi-dry apparatus under an electrical current of 15 mV, for 45 min. Then, membranes were incubated in 5% (*w*/*v*) dried skimmed milk powder in TBST solution for 1 h with gentle shaking at room temperature. The membranes were incubated with primary antibodies for phosphorylated-Akt (Santa Cruz Biotechnology, Inc., Dallas, TX, USA, #sc-7985), total-Akt (Santa Cruz Biotechnology, Inc., Dallas, TX, USA, #sc-8312), phosphorylated p38 (Cell signalling Technology, Danvers, MA, USA, #9211), total-p38 (Cell signalling Technology, Danvers, MA, USA, #8690), Bax (Santa Cruz Biotechnology, Inc., Dallas, TX, USA, #sc-493), Bcl-2 (Santa Cruz Biotechnology, Inc., Dallas, TX, USA, #sc-492), caspase 3 (Santa Cruz Biotechnology, Inc., Dallas, TX, USA, #sc-7148) and caspase-8 (Cell signalling Technology, Danvers, MA, USA, #9476) (all of the primary antibodies were diluted at 1:1000 in 1% (*w*/*v*) skimmed milk + TBST buffer) and horseradish peroxidase-conjugated secondary antibody (goat anti-rabbit (Merck, Darmstadt, Germany, #AP132P) and rabbit anti-goat (Merck, Darmstadt, Germany, #AP106P), which were diluted at 1:5000 in 1% (*w*/*v*) skimmed milk + TBST, after the heart protein was separated in SDS-PAGE. The band intensity quantitation was captured and analyzed by ChemiDoc XRS+, Image LabTM 2.0 Software (Bio-Rad Laboratories, Inc., Hercules, CA, USA). [24]

### 2.12. Statistical Analysis

All values were expressed as mean ± S.EM. All comparisons involving more than one group were assessed for significance using one-way analysis of variance (ANOVA), followed, when appropriate, by the Tukey–Kramer test. A *p*-value of less than 0.05 was considered significant.

## 3. Results

### 3.1. The Animal Body Weight and Heart Weight of Experimental Animals

**Figure 2:**
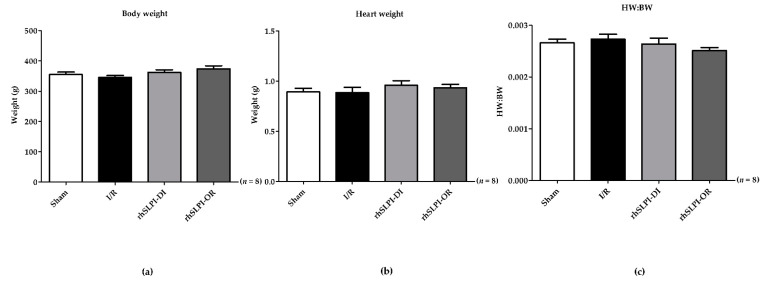
Determination of body weight of experimental animal before performing the surgical procedure of myocardial I/R injury (**a**), heart weight harvested at the end of the surgery (**b**) and heart weight to body weight ratio in rats of the study cohort (**c**). 3.2. Effect of Post-Ischemic rhSLPI Treatment on Infarct Size.

The animal body weight was measured before the surgery and heart weight was measured after the end of surgical procedures. The results show that the body weight of all groups was not significantly different (355.1 ± 8.21 g, 346.2 ± 5.77 g, 362.6 ± 8.544 g and 374.1 ± 10.31 g, *p* > 0.05) (Figure 2a). Similarly, the heart weight of rats in all groups was also not significantly different (0.893 ± 0.03 g, 0.886 ± 0.05 g, 0.960 ± 0.04 g and 0.9335 ± 0.03 g, *p* > 0.05) (Figure 2b). Therefore, the calculated heart weight and body weight ratio (HW/BW) also showed no significant difference in every group (0.0026 ± 0.0006, 0.0027 ± 0.0009 vs. 0.0026 ± 0.0001 and 0.0025 ± 0.0005, *p* > 0.05) (Figure 2c).

**Figure 3:**
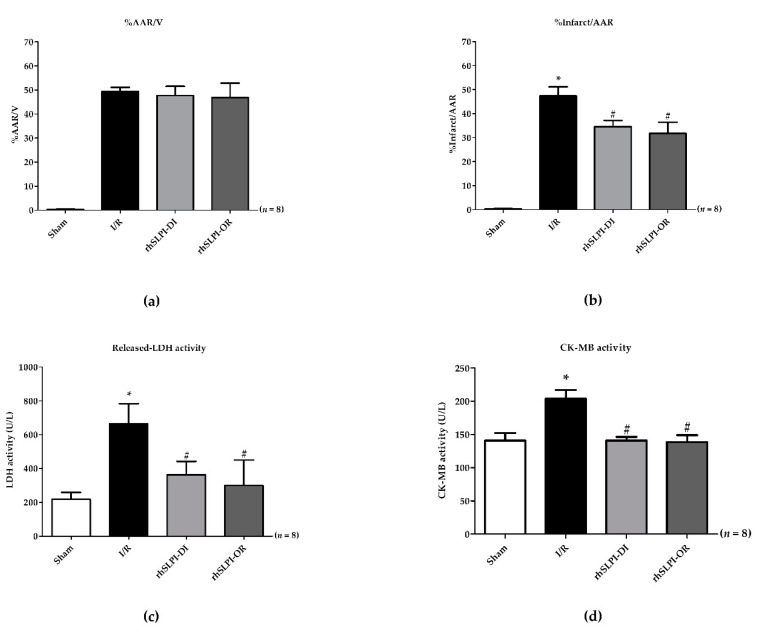
Determination of the percentage of area at risk (AAR) to ventricle volume (V) (**a**), percentage of infarct size to area at risk (**b**), activity of Lactate Dehydrogenase (LDH) (**c**) and Creatine kinase-MB (CK-MB) (**d**) (* *p* < 0.05 vs. sham) (^#^ *p* < 0.05 vs. I/R) (*n* = 8).

First, we confirmed by evaluating the area at risk (AAR) that there was no difference in the I/R injury induced in all rats. Indeed, AAR was similar among the study groups (Figure 3a).

The results in Figure 3b show that infarct size (% infarct/AAR) in rats treated by rhSLPI was significantly lower than that of the non-treated I/R group (34.53 ± 2.61% for rhSLPI-DI and 31.75 ± 4.64% for rhSLPI-OR vs. 47.32 ± 3.83% for IR, *p* < 0.05) (Figure 3b).

### 3.2. Effect of Post-Ischemic rhSLPI Treatment on Cardiac Biomarkers

Serum cardiac biomarkers were assessed by measuring both LDH and CK-MB activity.

The results show that the LDH activity of the I/R group was significantly higher than that of the sham group (664.9 ± 118.9 vs. 218.4 ± 40.79 unit per liter (U/L), *p* < 0.05) (Figure 3c). Treatment with rhSLPI significantly reduced serum LDH activity in both the rhSLPI-DI group and the rhSLPI-OR group compared to that of the I/R group (363.0 ± 79.91 U/L, 299.8 ± 150.2 U/L vs. 664.9 ± 118.9 U/L, *p* < 0.05) (Figure 3c).

Similarly, for CM-MB activity, values obtained for the I/R group were significantly higher than those of the sham group (203 ± 13.43 U/L vs. 14.8 ± 11.20 U/L, *p* < 0.05) (Figure 3d). Treatment with rhSLPI significantly reduced serum CK-MB activity in both treated groups vs. the I/R group (141.0 ± 5.27 U/L and 138.8 ± 10.08 U/L vs. 203.8 ± 13.41 U/L, *p* < 0.05) (Figure 3d).

### 3.3. Effect of rhSLPI Treatment on LVP in I/R Rat

The LVP of the I/R rat was evaluated by using high-fidelity micromanometer catheters placed inside the left ventricular chamber. The results were divided into three phases including at baseline (after stabilization for 30 min prior to LAD ligation), ischemia (30 min after LAD ligation) and reperfusion (at 120 min after reperfusion).

At the baseline phase, all of the LVP parameters including the EDP, ESP, dP/dt max, CtrI, dP/dt min, Tau/e, devP and HR were not significantly different when compared among groups (Table 1).

The LVP parameters of the ischemic phase (30 min after LAD ligation) are shown in Table 2. The result shows that ischemia induced a reduction in ESP, dP/dt_max_ and devP and an increase in EDP, dp/dt_min_ and Tau/e. Treatment with rhSLPI during ischemia significantly reduced EDP, dp/dt_min_ and Tau/e and increased ESP, dP/dt_max_ and devP.

The LVP parameters in the reperfusion phase (at 120 min after reperfusion) are shown in Table 3. The result shows ischemia induced a reduction in ESP, dP/dt_max_ and devP and an increase in EDP, dp/dt_min_ and Tau/e. Treatment with rhSLPI both during ischemia and at the onset of reperfusion significantly reduced EDP, dp/dt_min_ and Tau/e and increased ESP, dP/dt_max_ and devP.

### 3.4. Effect of Post-Ischemic rhSLPI Treatment on Inflammatory Cytokines Level

**Figure 4:**
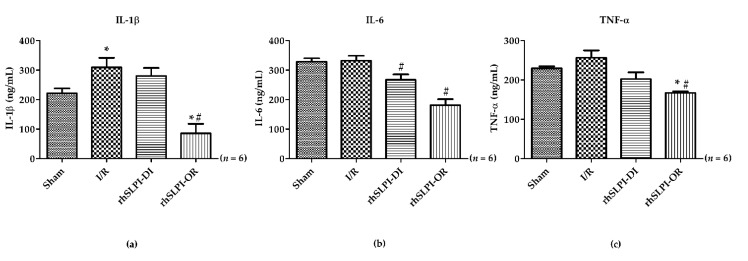
Determination of effect of post-ischaemic treatment of rhSLPI on cardiac inflammatory cytokines level including IL-1β (**a**), IL-6 (**b**) and TNF-α (**c**) in the sham group, I/R group, rhSLPI-DI and rhSLPI-OR (* *p* < 0.05 vs. sham) (^#^ *p* < 0.05 vs. I/R) (*n* = 6).

Excessive inflammation has been described as one of the pathophysiological mechanisms that occurs in myocardial I/R injury and plays a key role in inducing cell death [20]. In this study, the cardiac inflammatory cytokines level including IL-1β, IL-6 and TNF-α was measured by ELISA. The results show that there was a marked increase in the cardiac IL-1β level in the I/R group vs. the sham group (309.3 ± 32.45 ng/mL vs. 221.4 ± 16.91 ng/mL, *p* < 0.05) (Figure 4a). The rhSLPI treatment at reperfusion was able to drastically decrease the IL-1β level while rhSLPI treatment during ischemia was not. There was no difference in the I/R group vs. sham for the cardiac levels of IL-6 and TNF-α (IL-6: 332.1 ± 16.72 ng/mL vs. 328.9 ± 11.45 ng/mL, and TNF-α: 256.7 ± 18.68 ng/mL vs. 229.9 ± 5.02 ng/mL, *p* < 0.05) (Figure 4b,c). However, post-ischemic treatments with rhSLPI significantly reduced the cardiac IL-6 level when compared to the I/R group (267.3 ± 18.34 ng/mL vs. 332.1 ± 16.73 ng/mL, *p* < 0.05) (Figure 4b). Interestingly, the results show that cardiac IL-1β, IL-6 and TNF-α levels in the rhSLPI-OR group were significantly lower than the I/R group (Figure 4a–c).

### 3.5. Effect of Post-Ischemic rhSLPI Treatment on Oxidatively Modified Cardiac Proteins Level

**Figure 5:**
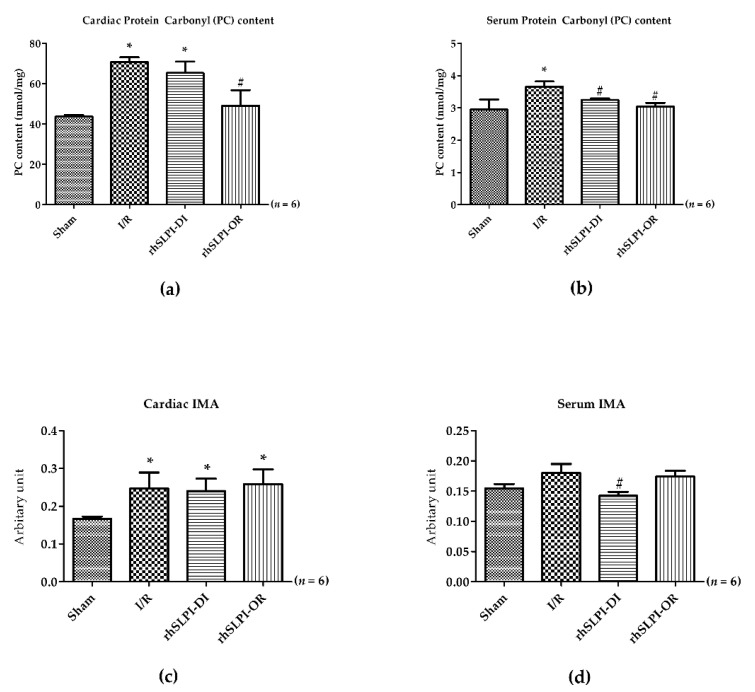
Determination of cardiac (**a**,**c**) and serum (**b**,**d**) protein modifications (protein carbonylation and ischemia-modified albumin). (* *p* < 0.05 vs. sham) (^#^ *p* < 0.05 vs. I/R) (*n* = 6).

The result shows that cardiac PC content levels in the I/R group were significantly higher than those of the sham group (cardiac PC: 70.75 ± 2.40 nmol/mg vs. 43.70 ± 0.78 nmol/mg, and serum PC: 3.66 ± 0.15 vs. 2.95 ± 0.31, *p* < 0.05) (Figure 5a,b). Treatment with rhSLPI in rhSLPI at reperfusion was significantly lower than that of the I/R group (49.08 ± 75.70 nmol/mg vs. 70.75 ± 2.40 nmol/mg, *p* < 0.05) (Figure 5a). In addition, the result shows that the serum PC content level in rhSLPI-DI and rhSLPI-OR was significantly lower than that of the I/R group (3.25 ± 0.04 nmol/mg and 3.04 ± 0.11 nmol/mg vs. 3.66 ± 0.15 nmol/mg, *p* < 0.05) (Figure 5b).

In addition, the results show that the cardiac IMA level in I/R, rhSLPI DI and rhSLPI-OR groups was significantly higher than that of the sham group (0.24 ± 0.04, 0.24 ± 0.03 and 0.24 ± 0.03 arbitrary unit (A.U.) vs. 0.16 ± 0.01 A.U., *p* < 0.05) (Figure 5c). However, the serum IMA level of the I/R group showed a trend to be greater than the sham group, but this was not statistically significant (0.17 ± 0.01 A.U. vs. 0.15 ± 01 A.U., *p* > 0.05) (Figure 5d); however, treatment with rhSLPI in the rhSLPI-DI group showed, in serum, a significantly lower IMA level than that of the I/R group (0.17 ± 0.01 A.U. vs. 0.15 ± 0.01 A.U., *p* < 0.05) (Figure 5d).

### 3.6. Effect of Post-Ischemic rhSLPI Treatment on Signal Transduction and Apoptosis Regulatory Molecules

**Figure 6:**
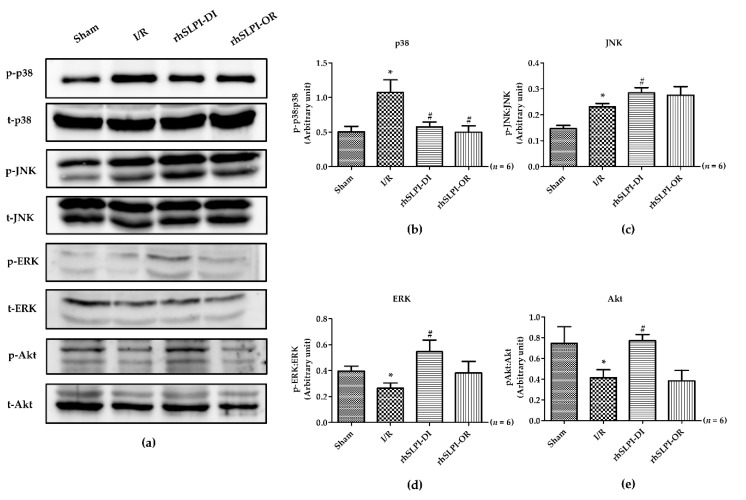
Determination of rhSLPI on activation of MAPK kinase involved in cell injury and survival in MI rat by Western blot analysis (**a**). The quantitative analysis for the MAPK phosphorylation was presented for p38 (**b**), JNK (**c**), ERK (**d**) and Akt (**e**). (* *p* < 0.05 vs. sham) (^#^ *p* < 0.05 vs. I/R) (*n* = 6).

To determine the effect of rhSLPI in signal transduction on the organ level response to myocardial ischemia/reperfusion injury, Western blot analysis was performed on the heart homogenate protein. The results show that there was a significant activation of p38 MAPK and JNK phosphorylation in the I/R group, when compared to the sham group (p38 MAPK: 1.07 ± 0.18 vs. 0.50 ± 0.07, and JNK: 0.23 ± 0.012 vs. 0.14 ± 0.01, *p* < 0.05) (Figure 6a–c). Similarly, ERK and Akt phosphorylation was significantly lower in the I/R group, when compared to the sham group (ERK: 0.26 ± 0.04 vs.0.39 ± 0.03, and Akt: 0.41 ± 0.07 vs. 0.74 ± 0.15, *p* < 0.05) (Figure 6a,d,e).

**Figure 7:**
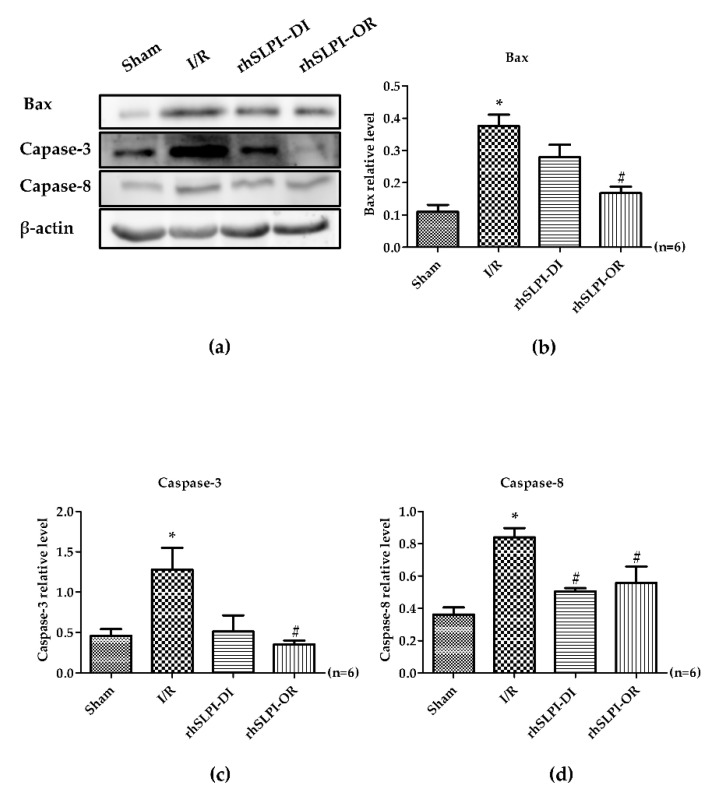
Determination of rhSLPI on apoptosis regulatory protein levels in MI rat by Western blot analysis (**a**). The quantitative analysis for the expression was presented for bax (**b**), caspase-3 (**c**) and caspase-8 (**d**) (* *p* < 0.05 vs. sham) (^#^ *p* < 0.05 vs. I/R) (*n* = 6).

In addition, there was a significant increase in Bax, caspase-3 and caspase-8 levels of the I/R group, when compared to the sham group (Bax 0.37 ± 0.03 vs.0.11 ± 0.02, caspase-3 1.27 ± 0.27 vs. 0.45 ± 0.08 and caspase-8 0.83 ± 0.05 vs. 0.36 ± 0.04, *p* < 0.05) (Figure 7a–d).

The post-ischemic rhSLPI treatment, in both treatment groups, showed that rhSLPI could significantly reduce p38 MAPK activation and caspase-8 (0.57 ± 0.06 vs. 1.07 ± 0.18 and 0.50 ± 0.01 vs. 0.83 ± 0.05, *p* < 0.05) (Figure 6b and Figure 7d). In contrast, rhSLPI could enhance JNK, ERK and Akt phosphorylation when compared with the I/R group (0.28 ± 0.01 vs. 0.23 ± 0.01, 0.54 ± 0.09 vs. 0.26 ± 0.04 and 0.77 ± 0.06 vs. 0.41 ± 0.07, *p* < 0.05). Moreover, treatment of rhSLPI at the onset at reperfusion showed significantly reduced activation of p38 MAPK, Bax, caspase-3 and capase-8 (0.49 ± 0.09 vs. 10.7 ± 0.18, 0.16 ± 0.01 vs. 0.37 ± 0.03, 0.35 ± 0.04 vs. 1.27 ± 0.27 and 0.55 ± 0.10 vs. 0.83 ± 0.05, *p* < 0.05) (Figure 6b and Figure 7b–d).

## 4. Discussion

In the present study, we demonstrated, for the first time, the cardioprotective effect of recombinant human secretory leukocyte protease inhibitor (rhSLPI) administered as a post-ischemic treatment in an in vivo rat model of myocardial I/R injury. The major findings of this study are that post-ischemic treatment with rhSLPI induced a reduction in infarct size, cardiac inflammatory cytokines levels and the production of oxidatively modified proteins and an improvement in cardiac function. Furthermore, rhSLPI treatment could associate with the attenuation of apoptosis regulatory signaling, such as p38 MAPK, Bax, caspase-3 and caspase-8, and the activation of cell survival protein Akt and ERK by phosphorylation.

Proteases are crucial for the maintenance of cellular homeostasis through the destruction of adverse misfolded or malfunctioning proteins, extracellular matrix (ECM) degradation and activation of cellular processes [25,26]. In cardiomyocytes, protease enzymes are basically active but counterbalanced by their endogenous inhibitors in order to finely control their action [27]. During myocardial I/R injury, the regulatory mechanisms are altered and they result in a large increase in both intracellular and extracellular protease activities [25,26,28,29,30,31]. The leukocyte-derived inflammatory serine proteases (ISPs), both from neutrophil- and mast cell-derived serine proteases, such as calpain, matrix metalloproteinases (MMPs) and cathepsins, have been reported to be involved in cardiac injury [26]. Alterations of protease activity not only cause cardiac cell injury and death but also result in subcellular cardiac remodeling and the loss of cardiac functions. Therefore, inhibition of protease activity can, therefore, be considered as a powerful strategy for preventing the expansion of injured tissue as well as the progression of cardiac remodeling and hypertrophy.

A strategy to inhibit protease activity, by using chymase inhibitor monotherapy, has been studied in several models, such as rodent, dog and pig, which provide similar information on reducing infarct size [32,33,34,35]. In addition, implementation of a dual-inhibitor (cathepsin and chymase inhibitor, DCCI) also showed reduced infarction size [36]. This evidence convinces the cardioprotective potential of serine protease inhibition against myocardial I/R injury. One of the concerning issues is that the protease inhibitors reported in those previous studies were small molecule chemical inhibitors. The findings from a computational study revealed the existence of chymase inhibitors of putative off-targets that were identified through various structural and functional similarity analyses as well as with molecular docking studies [37]. This is a warning for possible adverse effects in the case of small molecule inhibitors. Alternatively, using broad endogenous anti-protease peptides, which normally are expressed and function in the body, could provide more reliable results and safety. Secretory leukocyte protease inhibitor (SLPI) is an endogenous anti-serine protease protein that is normally expressed in several mucous tissues. Several studies described its pharmacological properties including antimicrobial and antiviral [11], as well as anti-inflammatory [9] and antiapoptotic activities [38]. Our study demonstrates that the post-ischemic administration of rhSLPI as early as possible, even during ischemia, could reduce infarct size and improve cardiac function.

The ischemic condition impairs cardiac function by reducing hemodynamic parameters for both the systolic and the diastolic functions. Treatment with rhSLPI either after the coronary ligation (during ischemia) or at the onset of reperfusion improved hemodynamic functions. This result correlates with reduced infarct size. An in vivo post-ischemic treatment with rhSLPI, injected at both time points, could significantly reduce infarct size. This finding is similar to that of our previous studies in both in vitro and ex vivo models of global ischemia [15,16]. The dose of rhSLPI in the present study (50 µg per 350 g body weight) is comparable to that used in our previous study (1000 ng/mL) which also provided cardioprotective effects [16].

Reactive oxygen species (ROS) are one of the detrimental components of I/R injury [39]. ROS play a major role in inducing cell injury and cell death via directly damaging cell membranes and, indirectly, through the recruitment of inflammatory cells and activation of the pro-apoptotic pathways [40]. It has been reported that SLPI could reduce intracellular ROS production in an in vitro simulated ischemia/reperfusion injury [15,16,17,19,41]. In this study, assessment of antioxidative stress was performed by determining the oxidatively modified products, PC and IMA. It has been reported that PC was increased in patients with acute myocardial infarction [42] and heart failure [43]. In addition, the IMA level was elevated in patients with acute coronary syndrome in all subgroups, including non-ischemic chest pain (NICP), unstable angina (UA) or myocardial infarction (MI) [44]. Therefore, PC and IMA could be cardiac biomarkers for assessing the oxidative stress in myocardial I/R models. In this study, rhSLPI could reduce oxidative stress by lowering the protein carbonyl content level from both cardiac tissue and serum, which could explain the beneficial effect toward infarct size reduction. However, it seems that IMA levels were slightly modified by rhSLPI treatment. It is possibly due to the lower sensitivity of IMA in terms of modification as well as in terms of being a diagnostic marker. The previous study showed that, in comparison with PC, IMA showed lower diagnostic sensitivity and area under the receiver operating characteristic (ROC)for diagnosis of non-ST elevation myocardial infarction patients [22].

**Figure 8:**
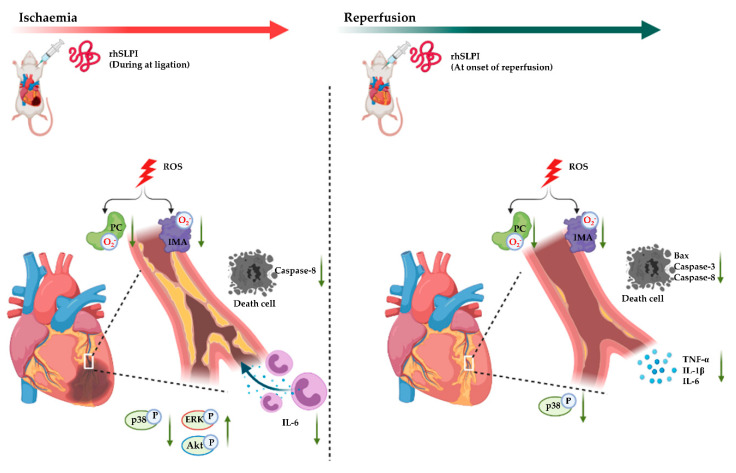
A schematic diagram of the major findings in this study. rhSLPI that was treated in the timing of the post-ischemia duration. rhSLPI could protect the heart from the ischemic condition. The mechanisms for this could be associated with the attenuation of intracellular ROS production, activation of Akt and p38 MAPK phosphorylation, reduction in inflammatory cytokines (TNF-α, IL-1β and IL-6) and reduction in apoptosis regulatory proteins (Bax, caspase-3, caspase-8).

One of the possible major effects leading to decreased cardiac injury, for the post-ischemic rhSLPI treatment, is the ability to reduce the reperfusion-induced inflammation. Accordingly, the results of the present study show that post-ischemic rhSLPI treatment could reduce production of inflammatory cytokines IL-1β, TNF-α and IL-6. The anti-inflammatory effect of SLPI was demonstrated in the present study, corroborating the results obtained by Jin et al. showing that SLPI reduced inflammatory cytokines in activated macrophages [45,46]. The attenuation of inflammation, as well as the infarct size decrease, could also be associated with the reduction in p38 MAPK phosphorylation in the rhSLPI-treated groups. Although p38 MAPK and JNK are both major signaling pathways known to be involved in the inflammatory response and cardiac cell death in I/R injury [47], post-ischemic treatment with rhSLPI could only reduce p38 MAPK phosphorylation, not JNK. Our previous report also demonstrated in vitro that rhSLPI treatment showed the association with pro-survival kinase activation, i.e., protein kinase B (PKB) or Akt [15,16]. Akt is a serine/threonine protein kinase, which is involved in survival pathways similar to p44/p42 MAPK and ERK1/2. Similar to our previous findings, our study here demonstrates that an in vivo post-ischemic treatment of rhSLPI could restore Akt and ERK1/2 phosphorylation, which consequently attenuates apoptosis regulatory molecules, e.g., Bax, caspase-3 and caspase-8. Taken together, the findings of our present study are consistent with our previous reports, using an in vitro and an ex vivo study model. Indeed, rhSLPI treatment induced cardioprotection by reducing infarct size, by attenuating cardiac inflammation oxidative stress and by its association with the regulatory molecules involved in cell death and injury, in order to finally result in an improved cardiac function (Figure 8).

There are some limitations in the current study that need to be addressed. The major source of protease secretion in myocardial I/R injury is from inflammatory cells in the heart after reperfusion, particularly neutrophiles [4]. In this study, cardiac tissue inflammatory cytokines were measured from a ventricular tissue homogenate, which is believed to contain a certain number of leukocytes in the cardiac tissue. Therefore, the inflammatory cytokines level shown in this study is actually from both ventricular tissue and residence leukocytes. The immunohistochemistry on the ventricular tissue section to determine or quantify infiltrated inflammatory cells needs to be investigated. Furthermore, assessment of protease activity, particularly cathepsin G and chymase, from ventricular tissue extract should also be performed by an in vitro cleavage of a fluorescent peptide substrate [36]. It is noteworthy that although our current study showed that treatment of rhSLPI could reduce p38 MAPK phosphorylation and activate Akt/ERK phosphorylation, this evidence could not provide a direct proof that SLPI protects the heart from I/R injury through those signaling pathways. The findings from this study, as well as the previous reports, could only suggest the association of those cell survival pathways with the protective effect of SLPI. Therefore, abolishing the protection, by using inhibitors of both pathways, could confirm the involvement of cell survival pathways on cardioprotection. It is also suggested that, for further investigation, the effect of long-term treatment of rhSLPI in myocardial infarction and post-myocardial infarction cardiac remodeling and heart failure progression should be assessed to provide the useful data before moving forward to clinical trials.

## 5. Conclusions

Our study demonstrates, for the first time, that rhSLPI treatment after coronary artery occlusion could provide cardioprotection by reducing I/R injury in an in vivo rat model. These findings suggest that rhSLPI could provide clinical benefit when given prior to reperfusion in patients with acute myocardial infarction.

## Figures and Tables

**Table 1:** The baseline parameters of left ventricular pressure (LVP) determined the effect of rhSLPI on the LVP parameters in I/R (baseline).

Parameters	Group			
	Sham (*n* = 8)	I/R (*n* = 8)	rhSLPI-DI (*n* = 8)	rhSLPI-OR (*n* = 8)
EDP (mmHg)	4.87 ± 0.53	4.23 ± 0.16	4.43 ± 0.53	4.75 ± 0.29
ESP (mmHg)	113.8 ± 6.34	104.80 ± 9.31	119.2 ± 15.30	118.9 ± 17.25
dp/dt_max_ (mmHg/s)	5233 ± 359.2	5071 ± 391.1	6113 ± 575.4	5470 ± 287.1
CtrI	100.5 ± 2.02	106.9 ± 5.59	99.73 ± 7.59	100.4 ± 6.0
dp/dt_min_ (mmHg/s)	−4989 ± 383.2	−4526 ± 487.1	−5627 ± 831.8	−5116 ± 362.6
Tau/e (ms)	10.18 ± 0.21	11.03 ± 0.43	9.67 ± 0.83	9.34 ± 0.73
devP (mmHg)	109.4 ± 5.40	108.1 ± 9.85	115.8 ± 16.75	109.8 ± 12.96
HR (bpm)	431.5 ± 18.09	426.9 ± 20.09	416.4 ± 10.04	437.8 ± 13.66

**Table 2:** The parameters of left ventricular pressure of ischemic phase determined the effect of rhSLPI on LVP parameters in MI (30 min after ligation).

Parameters	Group			
	Sham (*n* = 8)	I/R (*n* = 8)	rhSLPI DI (*n* = 8)	rhSLPI-OR (*n* = 8)
EDP (mmHg)	5.36 ± 0.40	8.63 ± 1.55 *	3.65 ± 0.46 ^#^	7.29 ± 0.18
ESP (mmHg)	111.8 ± 10.1	81.81 ± 9.69 *	131.2 ± 9.08 ^#^	101.8 ± 17.0
dp/dt_max_ (mmHg/s)	5072 ± 725.1	3547 ± 415.7 *	5983 ± 373.3 ^#^	4520 ± 541.0
CtrI	97.63 ± 5.21	101.5 ± 6.60	100.6 ± 2.55	80.88 ± 21.69
dp/dt_min_ (mmHg/s)	−4185 ± 206.6	−3139 ± 93.69 *	−5646 ± 701.1 ^#^	−4565 ± 590.5
Tau/e (ms)	10.11 ± 0.46	12.38 ± 1.05 *	10.04 ± 0.37 ^#^	8.744 ± 0.22
devP (mmHg)	106.9 ± 9.78	83.47 ± 7.72 *	126.7 ± 12.82 ^#^	105.5 ± 8.85
HR (bpm)	429.3 ± 19.13	405.3 ± 24.52	390.1 ± 15.31	421.2 ± 12.75

* *p* < 0.05 vs. sham, ^#^ *p* < 0.05 vs. I/R.

**Table 3:** The parameters of left ventricular pressure of reperfusion phase determined the rhSLPI effect on LVP parameters in MI (120 min after reperfusion).

Parameters	Group			
	Sham (*n* = 8)	I/R (*n* = 8)	rhSLPI-DI (*n* = 8)	rhSLPI-OR (*n* = 8)
EDP (mmHg)	5.85 ± 0.60	7.63 ± 0.08 *	2.871 ± 0.19 ^#^	4.084 ± 0.60 ^#^
ESP (mmHg)	105.6 ± 6.65	82.74 ± 6.63 *	111.1 ± 10.01 ^#^	94.47 ± 8.80 ^#^
dp/dt_max_ (mmHg/s)	4509 ± 527.4	2836 ± 373.4 *	4739 ± 466.8 ^#^	4458 ± 376 ^#^
CtrI	91.27 ± 5.79	94.41 ± 9.65	90.42 ± 2.79	72.85 ± 20.30
dp/dt_min_ (mmHg/s)	−4890 ± 569.3	−2418 ± 669.7 *	−4127 ± 244.6 ^#^	−3990 ± 264.3 ^#^
Tau/e (ms)	11.24 ± 0.53	14.83 ± 3.58	9.73 ± 0.54	8.754 ± 1.37
devP (mmHg)	104.4 ± 7.40	80.96 ± 7.25	96.28 ± 4.08	95.51 ± 6.61
HR (Bpm)	408.5 ± 20.81	373.9 ± 42.74	402.7 ± 6.47	410.3 ± 10.92

* *p* < 0.05 vs. sham, ^#^ *p* < 0.05 vs. I/R.

## Data Availability

The data presented in this study are available on request from the corresponding author.
